# Neural stem cell-derived exosomes and regeneration: cell-free therapeutic strategies for traumatic brain injury

**DOI:** 10.1186/s13287-023-03409-1

**Published:** 2023-08-08

**Authors:** Lin Zhong, Jingjing Wang, Peng Wang, Xiaoyin Liu, Peng Liu, Xu Cheng, Lujia Cao, Hongwei Wu, Jing Chen, Liangxue Zhou

**Affiliations:** 1 Department of Hematology, The First Affiliated Hospital of Chengdu Medical College, Chengdu, 610500 Sichuan China; 2 Department of Neurosurgery, West China Hospital, West China Medical School, Sichuan University, Chengdu, 610041 Sichuan China; 3 Tianjin Key Laboratory of Neurotrauma Repair, Institute of Neurotrauma Repair, Characteristic Medical Center of People’s Armed Police Forces, Tianjin, 300162 China; 4 Department of Health Management, Tianjin Hospital, Tianjin, 300211 China; 5 Department of Anesthesiology, West China Hospital, Sichuan University, Chengdu, 610064 Sichuan China

**Keywords:** Neural stem cell, Exosomes, Traumatic brain injury, Neuroregeneration, Neuroinflammation

## Abstract

Regenerative repair of the brain after traumatic brain injury (TBI) remains an extensive clinical challenge, inspiring intensified interest in therapeutic approaches to explore superior repair strategies. Exosome therapy is another research hotspot following stem cell alternative therapy. Prior research verified that exosomes produced by neural stem cells can participate in the physiological and pathological changes associated with TBI and have potential neuroregulatory and repair functions. In comparison with their parental stem cells, exosomes have superior stability and immune tolerance and lower tumorigenic risk. In addition, they can readily penetrate the blood‒brain barrier, which makes their treatment efficiency superior to that of transplanted stem cells. Exosomes secreted by neural stem cells present a promising strategy for the development of novel regenerative therapies. Their tissue regeneration and immunomodulatory potential have made them encouraging candidates for TBI repair. The present review addresses the challenges, applications and potential mechanisms of neural stem cell exosomes in regenerating damaged brains.

## Background

Exosomes are phospholipid bilayer membrane structures with a 40–100 nm diameter that are secreted from almost all mammalian cells. Initially, researchers believed that exosomes are waste products released by cells, but subsequent studies found that information exchange between cells is closely related to exosomes [1–3]. Exosomes can contain quite a few biologically active components such as lipids proteins and nucleic acids and are able to mediate long-distance communication from cell to cell, exchange genetic information, transmit immune signals and even function as a completely independent metabolic unit [4–6]. Numerous reports have shown that exosomes are involved in the operation of vital cellular physiological activities, especially involving the immune response [7–9], apoptosis [10–12], angiogenesis [13–16] and the inflammatory response [17–20], and they have become potential substitutes for cell-based therapies for various diseases, early diagnostic markers and targeted drug carriers.

As the “headquarters” of human functional activities, the regeneration and repair of the brain after injury is a problem in today’s scientific community. The fact that brain neurons have the genetic characteristic of being difficult to regenerate and the inhibitory microenvironment after traumatic brain injury (TBI) are key difficulties in neurological repair. Therefore, the focus of functional recovery after TBI is based on promoting neuroregeneration after injury and creating a suitable microenvironment. Growing evidence has proven that the use of stem cell-derived exosomes can confer more benefits in treating TBI than directly transplanting stem cells, creating new therapeutic pathways for nerve regeneration [21, 22]. Current cell-free therapeutic strategies for brain regeneration have been developed since exosomes were first discovered in sheep reticulocytes and named “exosomes” by Johnstone in 1987 [23, 24]. There has been remarkable progress in the field, especially its application in the regenerative repair of TBI. Advances in our understanding of the biological effect of exosomes have had a major impact on the fields of regenerative medicine, where they can be widely used as effective mediators of cell signalling for brain repair. Research has confirmed that exosomes are critical for cell-to-cell communication inside and outside the brain, showing that exosomes not only transmit signals over short distances inside cells but are also widely spread throughout the brain by cerebrospinal fluid.

Neural stem cells (NSCs) are self-renewing, differentiating and proliferating cell populations that can improve neurological function by stimulating vascular regeneration and neuroregeneration and repair. Current sources of neural stem cells fall into two categories, each with its own strengths and limitations. The first includes endogenous neural stem cells that are mainly distributed in the subventracular zone (SVZ) of the lateral ventricle and subgranular zone (SGZ) of the dentate gyrus of the hippocampus [25] and the second includes exogenous NSCs evolved from embryonic tissue. However, for both exogenous NSC transplantation and endogenous neural stem cell activation, there are deficiencies in the clinical application process. Exogenous neural stem cell transplantation has problems such as transplant adverse reactions and pretreatment safety screening tests, while the problems of low cell survival rate and poor differentiation to neurons of endogenous NSCs need to be further studied and discussed.

The complexity and practical issues of NSC treatment described above prompted us to explore other treatment strategies that can exert effects similar to those of NSCs. Because of the continuous deepening of stem cell research, growing evidence has demonstrated that cell substitution is not the primary mechanism of NSCs, but relies on the paracrine utility of NSCs [26]. NSCs release many neurotrophic factors through paracrine pathways[27, 28], including brain-derived neurotrophic factors (BDNF), glial cell line-derived neurotrophic factors (GDNF), neuronutrient-3, bioactive lipids and other paracrine factors, as well as extracellular vesicles, of which exosomes are the most representative [29]. As an important extracellular vesicle, NSC exosomes are responsible for vital biological functions such as cellular communication and material transport. Furthermore, they have the same biological functions as NSCs, playing a prominent role in tissue repair and regenerative medicine. While the mechanism of exosomes derived from NSCs is still unclear, it is undeniable that they open up a new research frontier for cell-to-cell communication. Exosomes produced by NSCs can enhance neuroplasticity, facilitate neuroregeneration and motor functional recovery after brain injury and provide therapeutic effects by modulating the function of neurons and glial cells in the local microenvironment and distant target cells [30]. Consequently, exosomes secreted by NSCs have great potential for clinical application and are a potential novel method for neurological diseases treatment. In this review, the basic characteristics and biological effects of neural stem cell exosomes are first described. Second, the role of exosomes secreted by NSCs in the diagnosis and treatment of TBI and its possible mechanism are summarized. Finally, the challenges of NSC exosomes as a new tool for TBI treatment are discussed.

### Overview of exosomes and neural stem cells

Exosomes are small vesicles secreted by cells that carry biological information and are produced via endosome and plasma membrane pathways. Exosomes can not only be used as biomarkers for the diagnosis and treatment of diseases, but also serve as a major medium of intercellular communication. In addition, exosomes play a critical role in the transport of membrane cells to cytoplasmic proteins, lipids and ribonucleic acid (RNA) [31]. The biogenesis of exosomes includes their contents, and subsequent pathways and mechanisms. NSCs can differentiate into neurons and glial cells to replace nerve cells that had been damaged or were depleted by injury. Therefore, NSCs could serve as a natural vigorous active resource and are believed to be a potential tool for treating TBI. Extensive research has found that NSC transplantation can significantly regenerate damaged tissues [32, 33]. Exosomes secreted by NSCs are believed to increase the advantage of stem cell-based treatment due to their ability to overcome the risks and difficulties of their parent NSC therapy. It has been proposed that NSC exosomes can penetrate the blood‒brain barrier (BBB) freely, inhibit apoptosis, mediate immune regulation, suppress inflammation and regulate autophagy. NSC exosomes as stem cell-free therapeutic strategies can be very promising candidates for TBI treatment by addressing the need for repair and regenerative therapy.

### Exosome contents and identification

Exosomes are rich in lipids, proteins, nucleic acids and other biologically active substances, which play a vital role in regulating cell physiological functions and affecting intercellular information transmission. It is generally believed that exosome components may differ depending on their origin and cell type. Exosomes have been identified as containing 4563 proteins, 764 miRNAs, 1639 mRNAs and 194 lipids [34]. Studies [35–37] have demonstrated that exosomes produced by different cells contain some of the same proteins, including three transmembrane proteins (CD9, CD81, CD63), polycystic synthesis-related proteins (Alix, TSG101), heat shock proteins (Hsp60, Hsp70, Hsp90), membrane transport and membrane fusion proteins (annexin, Rab protein), integrins, lipid-related proteins, etc. CD9, CD81 and CD63 are the most highly expressed exosome surface proteins and usually regulate adhesion, migration, invasion and fusion with receptor cells. Common proteins in exosomes involved in accelerating antigen entry into major histocompatibility complex (MHC) molecules are heat shock proteins (such as HSP70 and HSP90), which also act as assistants to help protein folding and intracellular transport [38]. Annexins and Rab proteins (Rab11, Rab27a, Rab27b) on exosomes might promote MVB fusion with cell membranes and secretion of exosomes [39]. In addition, there are costimulatory molecules such as CD86 and adhesion molecules such as CD54 and CD11b on the surface of exosomes [40]. Exosomes have a lipid bilayer structure, containing rich lipids such as acrylamide, cholesterol and sphingolipids. These lipids can maintain structural stiffness, protect the contents, maintain the stability of exosomes and facilitate long-distance transport [41]. Abundant genetic material is also contained in exosomes, such as mRNAs, miRNAs, lncRNAs and other noncoding RNAs, which are transmitted to receptor cells through exosomes to regulate proteins or serve as protein templates to regulate target cell differentiation, migration and other biological processes [42].

**Fig. 1:**
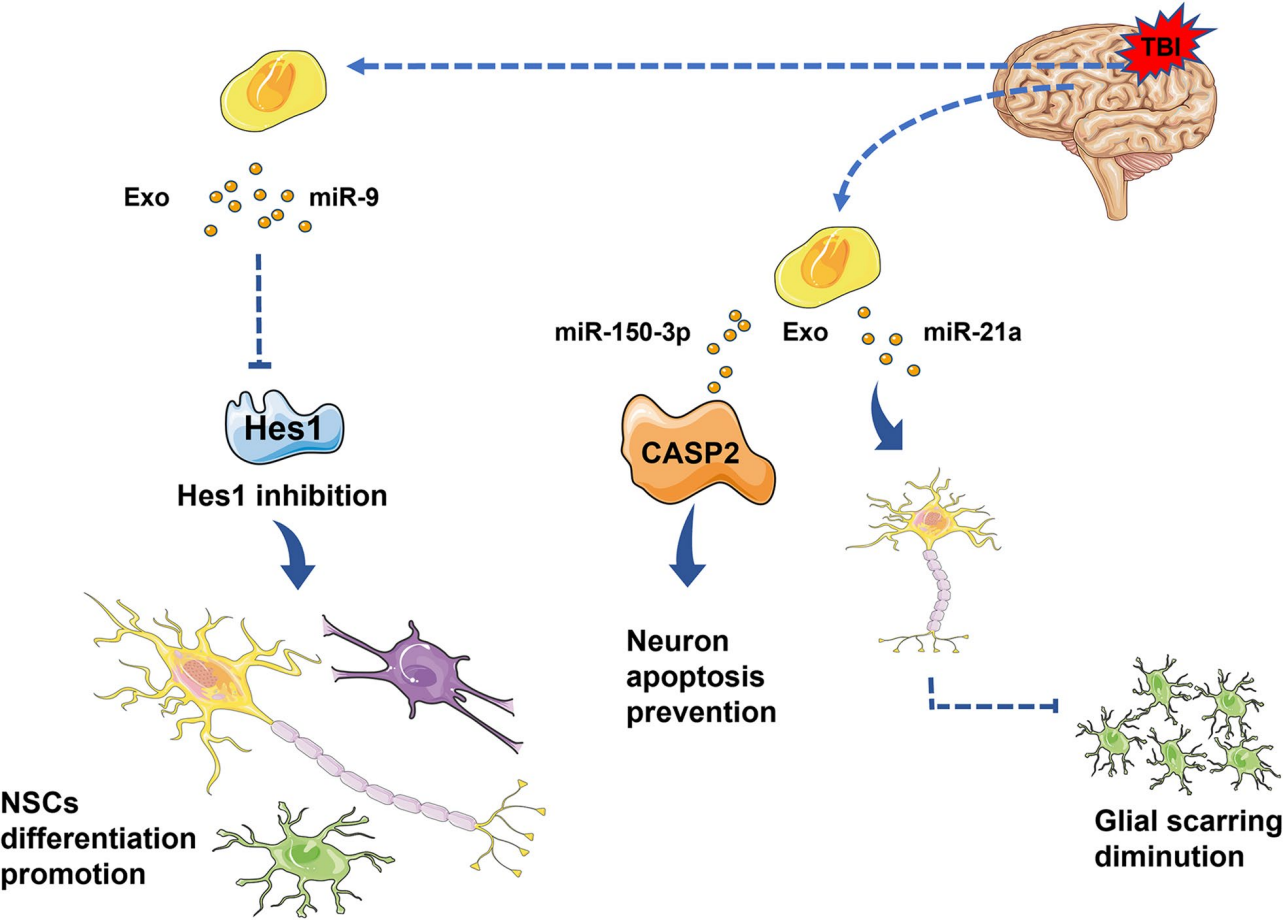
Schematic diagram of cellular communication between NSC exosomes and the surrounding microenvironment. NSC exosomes may exert their effects by acting on surrounding cells. For example, NSC-derived exosomes, especially miR-9, facilitate the differentiation of NSCs into neurons, oligodendrocytes and astrocytes by repressing Hes1. Moreover, miR-150-3p secreted by exosomes prevents neuronal apoptosis by targeting CASP2, or a high level of miR-21a can promote the differentiation of NSCs into neurons, which causes glial scar reduction

Notably, human neural stem cells (hNSCs) express c-MYC protein, a marker of NSCs, in addition to the proteins CD63 and CD81, which are common markers of exosomes. Second-generation sequencing showed that the miRNAs contained in hNSC exosomes were different from those from other sources. There were 113 kinds of miRNAs secreted by hNSCs, among which five kinds of miRNAs, including hsa-miR-1246, hsa-miR-4488, hsa-miR-4508, hsa-miR-4492 and hsa-miR-4516, were highly expressed [43]. As one of the highly expressed miRNAs, miR-1246 is a p53-targeting miRNA [44] that plays a crucial role in regulating cell development and apoptosis [45]. Another study showed that miR-21a is highly expressed in exosomes secreted by NSCs, which can promote the differentiation and regeneration of nerve cells [46] (Fig. 1).

We can identify exosomes by detecting surface marker proteins, morphology and particle size. Common methods for surface marker detection mainly include western blotting, enzyme-linked immunosorbent assay, immunofluorescence staining, flow cytometry analysis and mass spectrometry [47]. We usually use transmission electron microscopy, atomic force microscopy, scanning electron microscopy and freezing electron microscopy to identify the microstructure of exosomes. Usually, size-based characterization of exosomes can be identified by nanoparticle tracking analysis and dynamic light scattering. Based on the above methods, researchers mainly select several methods to identify exosomes. Zhang et al. combined transmission electron microscopy, nanoparticle tracking analysis and western blotting to identify exosomes secreted by neural stem cells [48]. In addition, Zhang et al. applied immunofluorescence staining, transmission electron microscopy and nanoparticle tracking analysis to identify exosomes derived from hypothalamic stem cells [49].

### Genesis pathway and mechanism of exosomes

**Fig. 2:**
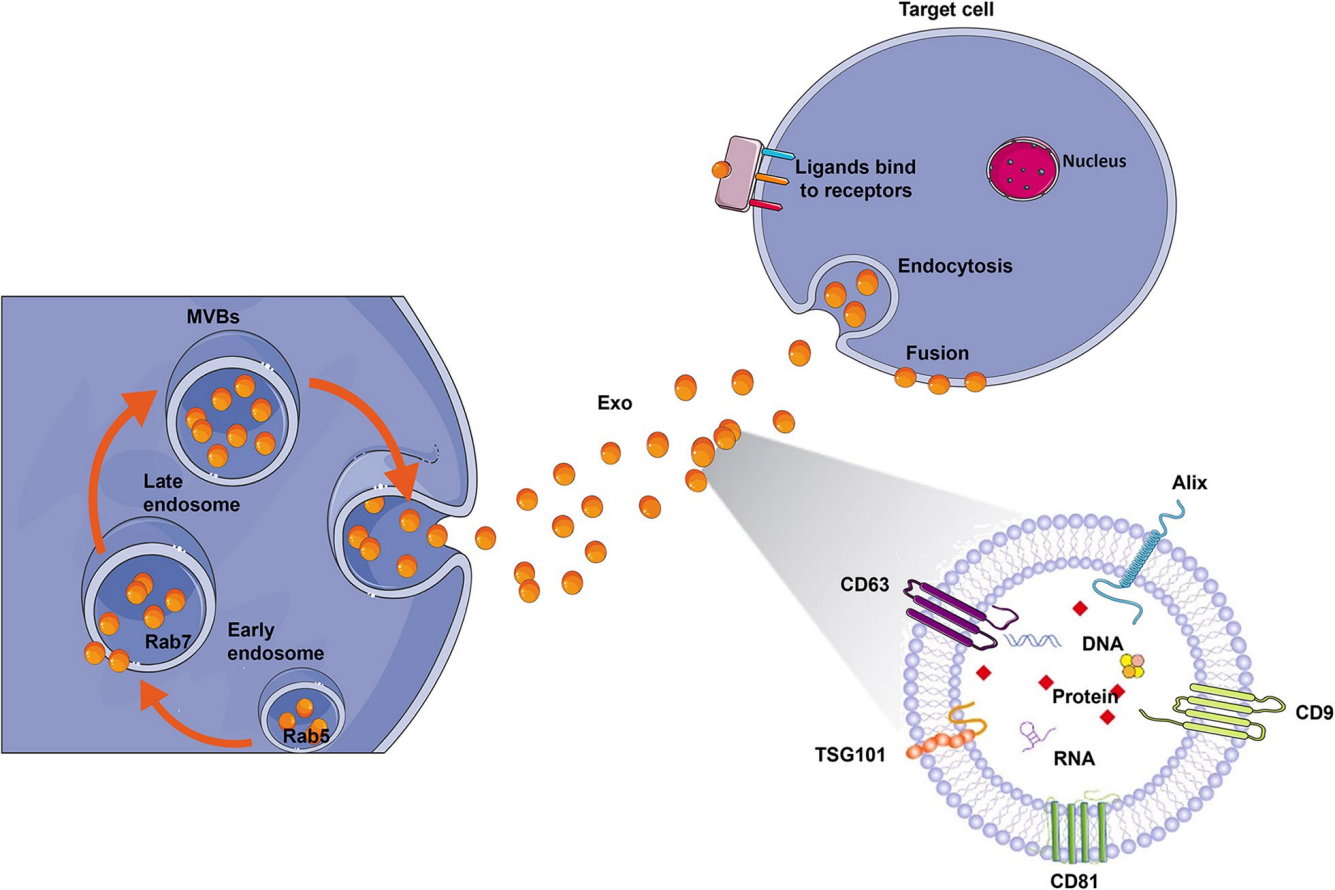
Schematic representation of exosome biosynthesis and ligand receptor binding. First, the cytoplasmic membrane, together with extracellular cargos, undergoes intracellular endocytosis from an early endosome. Late endosomes will next be generated through acidification and substance exchange of early endosomes. Late endosomes can eventually form MVBs. Ultimately, the multivesicular body will either fuse with lysosomes to degrade substances or fuse with the cell membrane to secrete exosomes into the extracellular space. Exosomes comprise a cargo of miRNAs, mRNAs, surface protein markers, including TSG101 and Alix, and the membrane markers CD9, CD63 and CD81. The released exosomes may affect the function of neighbouring and distant cells through several different mechanisms of action. One is that exosome membrane surface ligands bind to receptors on the receiving cell membrane, activating receptor-mediated signal transduction pathways, and the activated recipient cells ingest the contents into the cell through endocytosis. The other is that the receiving cell directly ingests exosomes into the cell in an endocytic manner, and the exosome contents are released into the cell. The last type is the direct fusion of the exosome membrane with the cell membrane, releasing the exosome contents into the cytoplasm

As a cardinal communication medium in cells, exosomes can bind to target cells in different pathways to transmit information. Currently, it is generally considered that the biogenesis of exosomes mainly involves plasma membrane and endosome pathways. In the endosome pathway, early endosomes bud from the plasma membrane to form early endosomes; subsequently, early endosomes germinate intraluminal vesicles (ILVs) to form multivesicular bodies (MVBs), and finally, MVBs fuse with the plasma membrane to release exosomes. In addition, some evidence has been corroborated that exosomes can bud directly from the plasma membrane, namely the plasma membrane pathway. Currently, the most studied biogenesis pathway of exosomes is the endosomal pathway. Cytoplasmic membrane invagination can form early endosomes, and early endosomes (Rab5 as a marker) mature to late endosomes (Rab7 as a marker) through acidification and material exchange. Late endosomes can finally form MVBs containing ILVs that are constituted by budding inwards depressions from the multivesicular membrane. Polyvesicles are degraded by fusion with lysosomes or autophagic lysosomes, and fusion with the cytoplasmic membrane results in the secretion of luminal vesicles outside the cell and ultimately forms exosomes with a diameter range from 40 to 160 nm (Fig. 2).

The endosomal pathway is currently the most widely studied mechanism of exosome genesis. Early endosomes gradually mature into MVBs, and the formation of MVBs has two mechanisms: endosomal sorting complex required for transport (ESCRT)-dependent and ESCRT-independent mechanisms. ESCRT, an essential machinery, comprises four various protein complexes: ESCRT-0, ESCRT-I, ESCRT-II, ESCRT-III and the associated AAA ATPase Vps4 complex [50]. The ESCRT-dependent mechanism to generate MVBs requires four steps: cargo aggregation, membrane invagination, cargo transport to newborn vesicles and vesicle neck splitting, and ESCRT is involved in the whole process [51]. Studies have shown that there is no substantial decrease in exosome biogenesis after the ESCRT mechanism is inhibited, suggesting that the ESCRT mechanism is not unique. Therefore, other reported exosome genesis pathways are classified into ESCRT-independent mechanisms. The ceramide (nSMase) pathway is the most important ESCRT-independent mechanism. For example, macrophages present antigens to dendritic cells through exosomes, and the transport of miRNAs from donor cells partly depends on the ceramide pathway.

### Purification and storage of exosomes

Due to their small size, low density and being mixed with similar components, the separation of exosomes poses a great challenge to their separation. Common separation methods of exosomes secreted by NSCs and other cells mainly include ultracentrifugation, ultrafiltration and size exclusion chromatography, polymer precipitation, immunoaffinity capture chromatography and microfluidic chip separation [52–54]. Among these methods, ultracentrifugation is the most generally used method and is currently regarded as the gold standard for exosome extraction. Moreover, different isolation techniques can also influence exosome bioactivity [55]. Therefore, standardized separation processes and quantitative methods are crucial for exosome quality and clinical application.

For the storage conditions of exosomes, it is also extremely vital to be able to maintain the integrity of exosomes in vitro. Some studies have suggested that different storage conditions may affect exosome size but do not have much impact on the cargo carried by exosomes. The data showed that exosome size decreased by approximately sixty percent after two days of storage at 37 °C. The exosome size remained basically unchanged after two days of storage at 4 °C, while the exosome size decreased significantly after 3 ~ 4 days. Interestingly, the morphology and size of exosomes can be maintained for one month when stored at − 20 °C [56]. Another study showed that exosomes washed twice with PBS can be stored for 1 ~ 7 days at 4 °C. After being resuspended in 10% dimethyl sulfoxide phosphate buffer solution, they can be stored at –80 °C for six months [57]. To date, there is still a lack of evidence that exosomes can be stored stably in vitro for a long time, so challenges in maintaining exosome integrity in vitro remain severe.

### Biological effects of NSC exosomes

Exosomes secreted by NSCs are involved in a wide range of interactions between neurons and microglia in the central nervous system [58]. Moreover, exosomes secreted by NSCs can transfer active substances from neurons to astrocytes, which may regulate astrocyte function, synaptic activity, neurovascular integrity and myelination. On the one hand, exosomes secreted by NSCs can carry specific particles from the original cells to target cells, leading to increased pathological changes, such as amyloid β-protein (Aβ) and tau protein β in Alzheimer’s disease (AD), prion proteins in spongiform encephalopathy and α-synuclein in Parkinson’s disease (PD). On the other hand, NSC exosomes can act as neuroprotective factors to promote peripheral nerve regeneration and nerve damage repair.

Exosomes derived from NSCs participate in the function and development of many physiological and pathological conditions of central nervous system diseases. NSC-derived exosomes are rich in specific miRNAs that can communicate with the microenvironment, mediate viral entry into cells and function as independent metabolic units [59]. Previous studies have shown that hNSC-derived exosomes contain 113 miRNAs. Notably, specific miRNAs can be transferred by hNSC secretions at the functional level, which is sufficient to mediate biological effects; for example, some of the miRNAs enriched in hNSC exosomes are vital to regeneration. As one of the highly expressed miRNAs of hNSCs, miR-4488 also exists in mesenchymal stem cells (MSCs) and participates in accelerating skeletal muscle regeneration [60]. Currently, the role of NSC exosomes as mediators of intercellular communication has gradually emerged. Cossetti [61] et al. revealed that NSC exosomes mediate communication with the microenvironment and that proinflammatory factors activate the interferon gamma (IFN-γ) signal transduction pathway in NSCs, resulting in specific components of the IFN-γ pathway acting on target cells via exosomes. NSC exosomes mediate viral entry into cells in a nonreceptor manner. The addition of NSC exosomes to coxsackie-adenovirus receptor (CAR)-deficient cells and adenovirus type 5 (Ad5) can transport Ad5 into CAR-deficient cells [62]. NSC exosomes can also serve as independent metabolic units, altering the concentration of key nutrients in the microenvironment and thereby affecting the physiological function of surrounding cells [63]. Furthermore, recent research has proven that exosomes derived from NSCs in the subventricular region of mice are rich in the miRNAs let-7, miR-9, miR-26 and miR-181, which specifically target microglia and affect their morphology, function and proliferation in the nervous system after regeneration [64, 65].

### Mechanisms underlying NSC exosome therapy for TBI

**Fig. 3:**
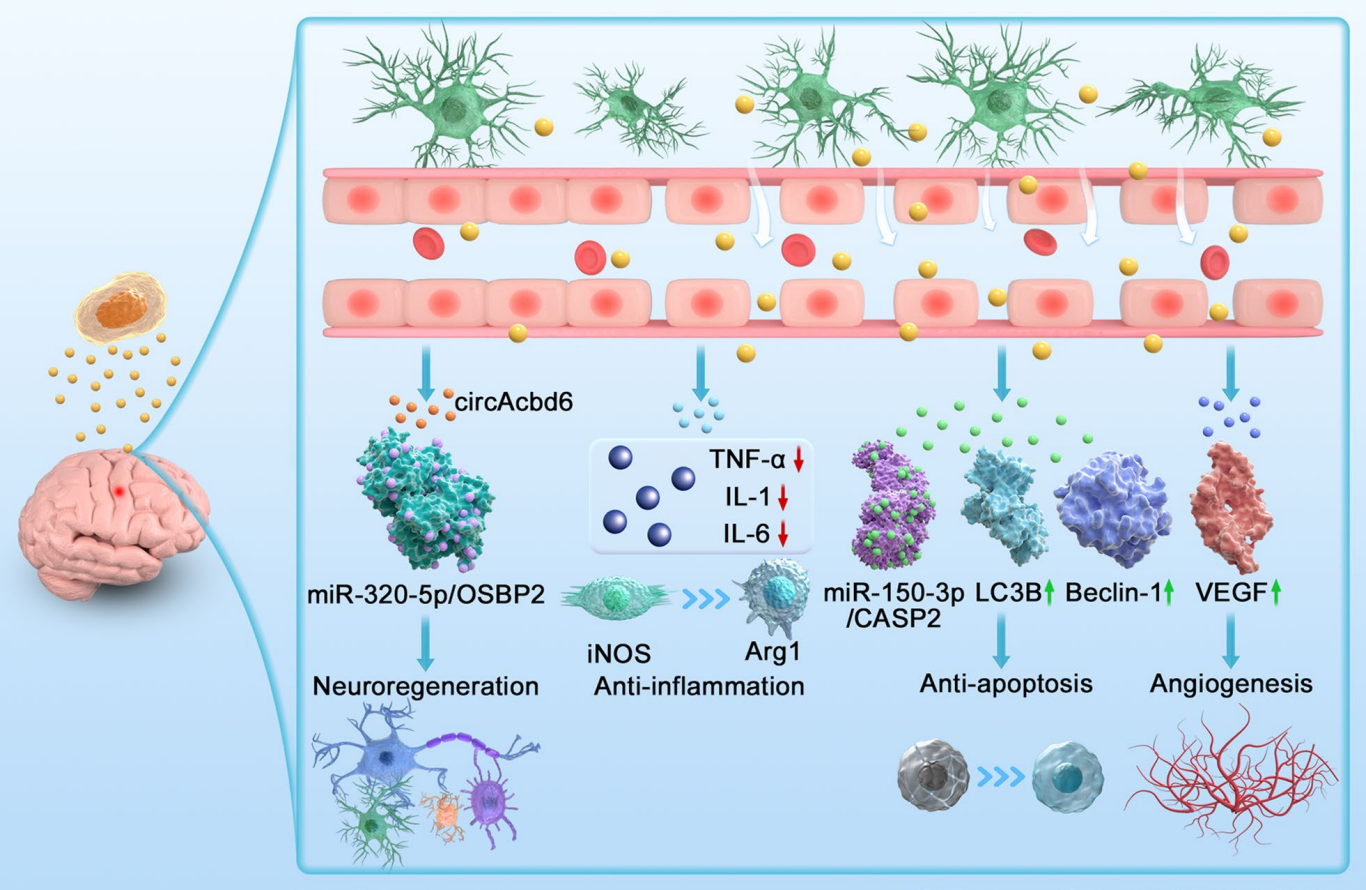
A schematic of the possible mechanisms of exosomes secreted by NSCs for TBI therapeutic effects. Exosomes secreted by NSCs contain numerous active ingredients of cells, can cross the BBB freely, inhibit the inflammatory response and neuronal apoptosis, promote neuroregeneration and angiogenesis, etc. In response to neuroregeneration, NSCs secrete exosomes that contain circAcbd6 and further promote the differentiation of NSCs into cholinergic neurons via the miR-320-5p/oxysterol-binding protein–related protein 2 axis. NSC-derived exosomes contain components that inhibit the inflammatory response, downregulate the expression of proinflammatory factors such as TNF-α, IL-1 and IL-6 and promote microglial polarization from the M1 to M2 phenotypes. Upon anti-apoptosis, exosomes secreted by NSCs transfer miR-150-3p, an enriched miRNA, and suppress neuronal apoptosis. Increased expression of the autophagy marker proteins LC3B and beclin-1 caused by NSC exosomes also reduced apoptosis of nerve cells. For the promotion of angiogenesis, neural stem cell exosomes can upregulate VEGF levels, which in turn accelerate angiogenesis. Schematic created with CINEMA 4D

A previous study demonstrated that NSCs are expected to become an efficient strategy for TBI treatment in both experimental models and clinical practice [66, 67]. Nevertheless, it has recently been proven that the main mechanisms by which NSCs are related to brain remodelling and functional recovery after TBI are not dependent on cell substitution utility but may rely on paracrine effects such as exosomes secreted from NSCs [68]. Recently, it has been confirmed that NSC exosomes, as carriers of signalling molecules, participate in mediating the communication of nerve cells in the pathological process after brain injury and take part in the occurrence and repair of post-TBI injuries by regulating the information communication between cells. Small RNA sequencing, proteomic analysis and validation of select miRNAs and proteins proved that exosomes of human iPSC-derived NSCs were enriched in miRNAs and proteins involved in pro-neuroregeneration, anti-apoptosis, anti-inflammation, antioxidant effects, blood‒brain barrier repair, etc. In addition, they also contain miRNAs and/or proteins that promote synaptic plasticity and enhance cognitive function recovery (Fig. 3).

### NSC exosomes promote neuroregeneration

The complex pathophysiological mechanism caused by TBI eventually leads to primary neuronal damage and delayed neuronal damage, which is mainly manifested in the programmed death of neurons.

In the brain, neurons, astrocytes, microglia, vascular endothelial cells, vascular perivascular cells, the basement membrane and the extracellular matrix make up the neuronal vascular units [69]. A series of cross-reactions with neural stem cells occur between neurovascular units and neural stem cells, which can carry out endogenous brain repair after TBI, including neurogenesis and vascular regeneration [70, 71]. Exosomes derived from NSCs play a prominent role in this process. As signal transmitters, exosomes mediate the physiological activities of target cells and promote the repair of TBI. The study suggested that circRNA Acbd6 (containing acyl-CoA binding domain 6) upregulated in exosomes of hippocampal NSCs promotes differentiation of NSCs into cholinergic neurons via the miR-320-5p/oxysterol-binding protein-related protein 2 axis and is targeted to mediate neurogenesis [72]. In this study, NSC-derived exosomes significantly enhanced the number of newborn neurons (stained for Tuj-1 and ChAT) detected in the hippocampus compared to the controls. NSC exosomes not only have a neurogenic effect on the hippocampus but also promote cell proliferation and enhance cell vitality. As reported in the study, the exosomal miR-210 produced by neural progenitor cells induced by hypoxia has a significant effect on cell proliferation [73]. Another study also showed that NSC-derived exosomes regulate and control NSC differentiation through the miR-9-Hes1 axis, which unveils a possible mechanism of exosome (Exo)-mediated NSC differentiation [74].

Research has revealed that exosomes secreted by NSCs are involved in a wide range of interactions between neurons and microglia in the CNS and participate in brain injury repair [58]. In addition, exosomes secreted by NSCs can transfer active substances from neurons to astrocytes in the brain, which may regulate the function of astrocytes, affecting synaptic activity, maintenance of neurovascular integrity and myelination. The main method of post-TBI repair is nerve and vascular regeneration, including the repair of the integrity of the BBB. Studies have examined the therapeutic effect of human NSC-derived exosomes on treating BBB leakage in vitro and confirmed that BBB disruption could be corrected by exosomes derived from NSCs. In addition, other studies have shown that miR-21a is highly expressed in exosomes secreted by NSCs, which can promote nerve cell differentiation and regeneration [46]. Furthermore, the intranasal administration of exosomes derived from hiPSC-NSCs led to exosome phagocytosis by neurons and neuroglial cells in all adult rat brain regions and enhanced hippocampal neurogenesis.

### NSC exosomes reduce the inflammatory response

Neuroinflammation has proven to be a crucial part of the pathophysiological process of TBI. Its pathological changes mainly include glial cell activation, upregulation of proinflammatory cytokines, increased inflammatory cell infiltration and increased blood and brain barrier permeability [75]. As inflammatory cells, leukocytes, microglia and astrocytes are involved in the pathological process of neuroinflammation. Various types of quiescent glial cells are activated including initiating microglial activation and continuing astrocytic activation after TBI [76]. Microglia, as macrophages of the nervous system, affect post-TBI neurologic function through dynamic polarization between proinflammatory microglia (M1) and anti-inflammatory microglia (M2) [77]. Astrocytes are activated during neuroinflammation, and a fraction of the activated astrocytes are called A1 astrocytes. A1 astrocytes exhibit a cytotoxic phenotype that is destructive to synapses and induces neuronal and oligodendrocyte apoptosis. Neuroinflammatory overreaction not only initiates a primary injury phase but also induces a prolonged secondary phase. Therefore, how to relieve acute neuroinflammation after TBI and establish new treatments for TBI is crucial in the future.

Inflammatory modulation could decrease the depletion of neurons and oligodendrocytes. Inflammation modulation by suppressing polarization towards proinflammatory microglia, inhibiting the activation of A1 astrocytes, facilitating anti-inflammatory microglial activity and enhancing apoptotic cell clearance may provide beneficial neuroprotection for TBI. Exosomes are important carriers of intercellular communication. Both microglia and neural stem cell-derived exosomes mediate inflammatory injury and exert neuroprotective effects. Microglia-derived exosomes affect NSC proliferation and differentiation through encapsulated proteins and miRNAs after brain injury [78]. Exosomes derived from NSCs could produce complex effects by crosstalk with microglia, such as inhibiting autophagy and promoting M2 microglia/macrophage polarization, significantly reducing the damaged area of brain injury. Increasing attention has been given to the mechanism of exosomes secreted from NSCs in treating TBI in recent years. Immunomodulatory therapies with exosomes derived from NSCs are a therapeutic strategy targeting TBI aimed at guiding the transition of inflammation towards the reparative phenotype. Research has confirmed that exosomes derived from hNSCs can significantly promote the recovery of motor function in TBI rats by inhibiting the expression of reactive astrocytes and enhancing the expression of doublecortin, thereby regulating neuroinflammation and promoting neurogenesis [79]. NSC-derived exosomes can serve as microglial morphogens, which might influence neuroinflammation in the adult brain [65]. Recently, studies have proven that systemic administration of cell-free exosomes generated by NSCs could promote microglial polarization from M1 to M2 phenotypes, which in turn reduces chronic inflammation [80]. Mehrdad Hajinejad et al. [81] proposed that hNSCs can downregulate the expression of Toll-like receptor 4 and its downstream signalling pathway, including NF-kβ and proinflammatory factors such as interleukin-1β. NSC exosomes can suppress neuroinflammation of cerebral ischemia with pathological processes similar to traumatic brain injury. NSC exosomes reduced the expression of inflammatory cytokines including TNF-α and IL-1β, while increased the expression of anti-inflammatory cytokine IL-10, which can better ameliorate elevated immune response after NSC transplantation, and promoted the motor function recovery [82]. Studies of the anti-inflammatory activity of exosomes derived from hiPSC-NSCs have shown that they suppress the expression of a proinflammatory cytokine [83]. In conclusion, exosomes generated by NSCs exhibit both anti-inflammatory and neuroprotective effects.

### NSC exosomes promote angiogenesis

Marshall et al. [84] found that 90% of TBI patients who died had ischaemic changes. Therefore, ischaemia is one of the main mechanisms of poor prognosis caused by secondary brain injury, such as brain oedema, cerebral infarction and even death. Secondary microvascular thrombosis leads to dysfunction of cells in the neurovascular unit, destruction of the BBB and injury to ischaemic tissue cells after TBI. Although the brain can spontaneously undergo limited remodelling during recovery from ischaemia, it is far from sufficient. Therefore, anchoring the material premise of neovascularization of tissues around brain injury, exploring strategies to promote angiogenesis after TBI and alleviating or even reversing ischaemia and hypoxia have aroused great concern. As a vital biological barrier of the brain, the BBB is used to maintain biological stability and provide a microenvironment for precise neural regulation [85, 86]. Cerebral microvascular endothelial cells are a key component of the BBB. Once the BBB is destroyed, numerous toxic, harmful and macromolecular substances enter the brain tissue, destroying the normal physiological balance of the brain. Therefore, repair of the BBB has become a target for the treatment of TBI.

It has been demonstrated that NSC transplantation can serve as a promising therapy for brain injury and especially exhibits proangiogenic and proregenerative effects [87]. Exosomes secreted by NSCs play an important role in the promotion of angiogenesis in brain injury. Vascular endothelial growth factor (VEGF) is known to play a cardinal role in the formation of blood vessels. Zhang et al. [48] found that neural stem cell exosomes can upregulate VEGF levels, which in turn promotes the formation of new blood vessels, accelerates angiogenesis and improves damaged neurological function after central nervous system injury. Exosomes of NSCs given after cerebral ischaemia‒reperfusion injury release neuroprotective factors, trophic factors and immunomodulatory factors. These factors promote endogenous neurogenesis, angiogenesis and the formation of new vascular networks, minimizing apoptosis and inflammation-induced damage [88]. Sun et al. [89] suggested that exosomes derived from hNSCs could improve motor function and reduce lesion area of male rats, along with increased migration of endogenous NSC to the lesion site. Moreover, administration of exosomes derived from hNSCs could enhance the vascular endothelial growth factor receptor 2 (VEGFR2) expression in male rats, which is of great significance for vascular regeneration after traumatic brain injury. Moreover, NSC exosome especially the proinflammatory factor interferon gamma treated exosomes showed increased microvascular density at the site of the cortex and striatum regions in brain ischemic injury model [48]. They may benefit from the abundant VEGF-A contained in NSC exosomes, which promotes microvascular regeneration. Angiogenesis and neurogenesis are interdependent in the neurovascular unit and endothelial cells involved in angiogenesis provide growth factors that regulate NSC self-renewal and neurogenesis [90]. Angiogenesis is not only beneficial to the repair of the BBB but also has an important impact on the nerve cells of ischaemic brain tissue. Angiogenesis can form a sufficient number of microcerebral blood vessels to improve blood flow in ischaemic tissue, improve microcirculation in ischaemic tissue and promote the repair of ischaemia‒reperfusion damaged nerve cells.

### NSC exosomes inhibit apoptosis

The cascade after TBI can cause apoptosis of nerve cells [91]. A series of changes after TBI include the proliferation of neurotoxic A1 astrocytes, the activation of cysteine-containing aspartate proteolytic enzyme (Caspase), the production of excitatory amino acids such as glutamic acid, the destruction of mitochondria, lipid peroxidation and calcium ion concentration imbalance. Studies have suggested that excessive apoptosis of nerve cells can further exacerbate inflammatory responses, vascular leakage, cerebral oedema, cerebral haemorrhage and hypoxia [92]. Oxidative stress and neuronal apoptosis are closely related to neurological impairment and cognitive dysfunction after TBI [93]. Current therapeutic interventions have shown neuroprotective effects of exosomes, which diminish reactive oxygen species (ROS) levels by delivering antioxidants to neurons and alleviating oxidative stress-mediated apoptosis [94, 95]. Therefore, the biological means by which exosomes inhibit nerve cell apoptosis are of great significance for the study of nerve tissue damage repair after TBI.

NSC exosome administration is an underlying strategy for suppressing apoptosis resulting from brain injury. Previous research has shown that neural stem cell-derived exosome administration can effectively reduce microglial density and neuronal apoptosis, thereby steadily improving functional recovery after middle cerebral artery occlusion [96]. Numerous miRNAs have been revealed to be expressed in NSC exosomes, some of which are believed to exert important impacts on diverse kinds of neural cell damage. It has been reported that NSC exosomes promote neuroprotective effects by transferring miR-150-3p, which is enriched in NSCs, thus suppressing neuronal apoptosis after brain injury [97]. In an vitro study, NSC exosomes inhibited apoptosis and promoted SH-SY5Y cell proliferation under both normal and oxygen–glucose deprivation culture conditions. In addition, in vivo research revealed that NSC exosomes significantly decreased the infarction area and suppressed the apoptosis of neurons after brain injury. Research has confirmed that the most abundant expressed miR-150-3p in neural stem cells exosomes exerts a protective function against hypoxic–ischemic brain injury by inhibiting the apoptosis of neural cells both in vivo and in vitro [97]. RONG et al. revealed that exosomes derived from NSCs promoted autophagosome formation by increasing the expression of the autophagy-tagged proteins LC3B and beclin-1, which in turn reduced nerve cell apoptosis, activated microglia and inhibited neuroinflammation by activating autophagy [81]. Another study has shown that injection of NSC-conditioned medium into an ischaemia‒reperfusion injury model effectively inhibited the area of cerebral infarction, maintained the ultrastructure of mitochondria at the ischaemic site and inhibited apoptosis of nerve cells by promoting the expression of Bcl-2. Additional studies have demonstrated that hNSC exosomes could inhibit neuronal apoptosis and enhance the nuclear transfer of Nrf2 in response to oxidative stress, in addition to promoting neuronal axon elongation and human umbilical vein endothelial cell (HUVEC) angiogenesis [98].

### Potential therapeutic effects of engineered neural stem cell exosomes targeting brain injury

Due to their unique nanostructure, lack of immunogenicity [99, 100], low toxicity [101, 102], and good permeability, with the ability to cross the BBB [103, 104], coupled with their important role in tissue regeneration, immune regulation and disease recognition, exosomes have great therapeutic potential in the treatment of central nervous system injury, but natural exosomes have poor targeting ability, which greatly reduces the therapeutic effect. Engineered exosomes have the ability to actively target specific cell types and tissues [105]. The development of nanotechnology has brought new hope for targeted drug delivery, and nanopreparations based on the structure and function of exosomes are expected to overcome central nervous system barriers, transfer neuroprotective molecules to the brain parenchyma and provide effective therapeutic means targeting brain regions for the treatment of central nervous system injury diseases.

**Fig. 4:**
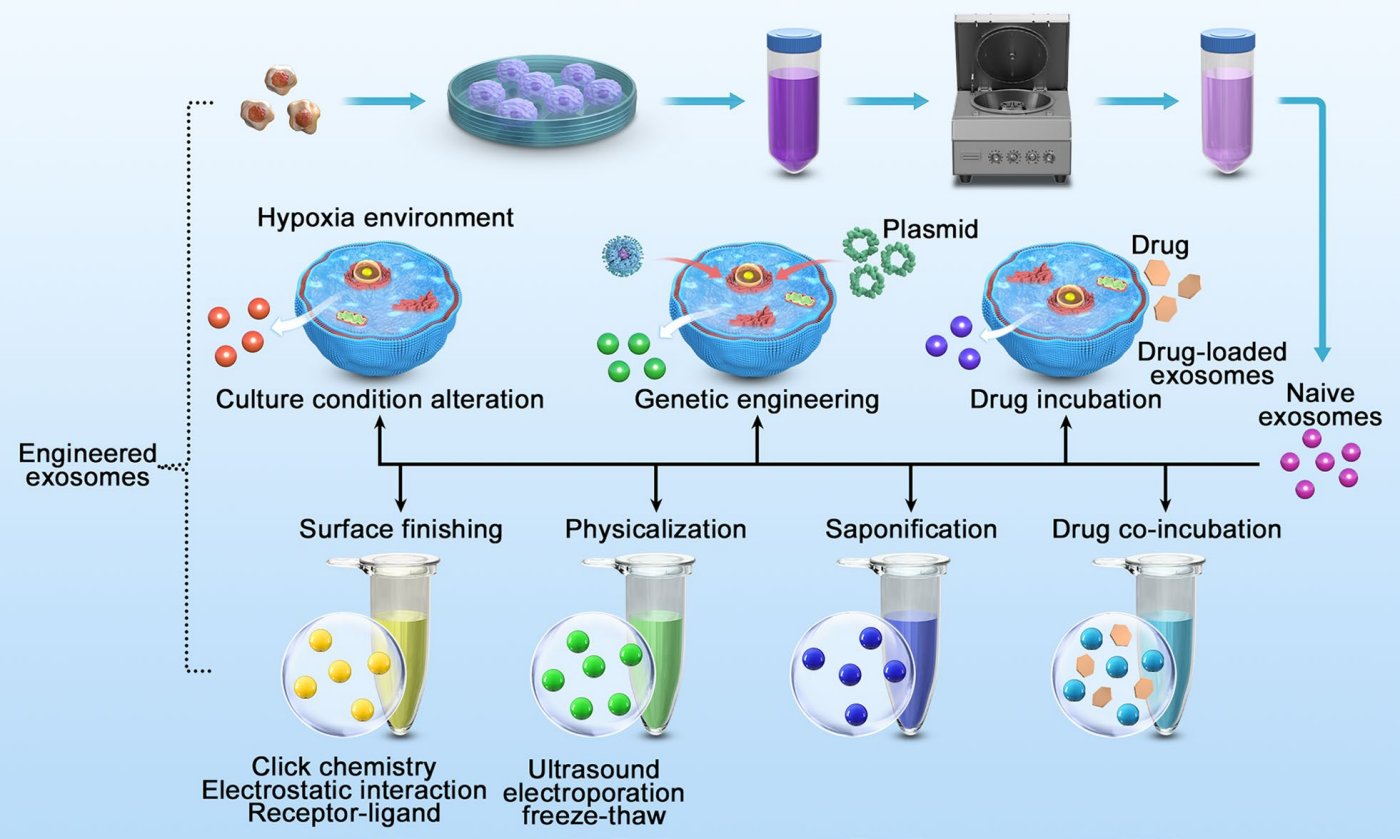
Preparation strategies for engineered exosomes: cell engineering and exosome engineering. Bioengineering of exosomal parent cells mainly includes genetic engineering, coincubation of parental cells with drugs and changes in cell culture conditions. Exosome engineering adopts specific techniques to directly modify the surface proteins or cargos of purified natural exosomes, including incubation, physical modification, saponification and surface modification. Among them, physical methods are divided into ultrasound, electroporation and freeze‒thaw, and surface modification is divided into covalent modification represented by click chemistry and noncovalent modification represented by electrostatic interactions and acceptor–ligand binding. Schematic created with CINEMA 4D

One of the strategies for the preparation of engineered exosomes is cell engineering. Genetic engineering of parental cells is one of the commonly used cell engineering techniques, that is, directly modifying exosomes during exosome biogenesis. Alvarez-Erviti et al. [106] discovered that exosomes secreted by dendritic cells transfected with plasmids fused with neuron-specific rabies virus glycoprotein and exosomal lysosomal-associated membrane protein could deliver siRNA to the brain in a targeted manner. Coincubation of parental cells with drugs is another commonly used method of engineered exosome preparation. For the first time, paclitaxel was directly incubated with bone marrow mesenchymal stem cells to target the proliferation of pancreatic cancer cells through exosome packaging and delivery of active drugs in vitro [107]. Another strategy for engineering exosomes is exosome engineering, that is, the modification of the surface proteins or contents of purified exosomes using specific techniques under appropriate conditions, which is mainly divided into incubation [108], physical methods [109], saponification [110, 111] and surface modification [112, 113]. Compared with the low efficiency and high difficulty of cell engineering, direct modification of purified natural exosomes can directly obtain a large number of engineered exosomes and reduce the uncertainty in the cell culture process, which is of great significance for the mass production of engineered exosomes (Fig. 4).

The utilization of well-characterized exosomes derived from NSCs under specific culture environments and NSC-derived exosomes engineered to carry the required miRNAs, mRNAs and proteins holds great promise in the treatment of brain injury. Neurons could be specifically targeted to receive a functional siRNA by systematically injecting engineered exosomes [114]. Chen et al. [115] demonstrated that engineered exosome BDNF-hNSC-Exos can increase the survival rate of neural stem cells in a hypoxic environment and promote the differentiation of neural stem cells into neurons. In this study, BDNF was loaded into hNSC-Exos to construct engineered BDNF-HNSC-Exos, which were injected into the ischaemic region of an animal model of ischaemic stroke by a stereotactic method. Subsequently, the authors demonstrated that BDNF-hNSC-Exos promote the differentiation of hypoxic neural stem cells into neurons. Additionally, BDNF-hNSCs-Exo significantly inhibited inflammation and reduced the volume of cerebral infarction after injection of BDNF-hNSCs-Exo into the brains of rats. Another study revealed that NSC-derived exosomes could be engineered for drug delivery to the brain [116]. In their study, NSCs are genetically modified to package a fluorescent protein in the interior of exosomes. The fluorescent protein was detected at the basolateral side of the BBB after incubation of the in vitro BBB with fluorescent protein-loaded exosomes, indicating that NSC-derived exosomes carry their cargo across the BBB. NSCs have also been engineered to continually generate exosomes containing therapeutic cargo at a high dose using a booster plasmid, providing a continuous therapeutic source following intracerebral implantation [117].

## Perspectives and challenges

Similar to most exosomes, exosomes secreted by NSCs have the following advantages in the treatment of brain injury. First, exosomes are nanosized vesicles that can cross the BBB smoothly and are easily absorbed, with a very low likelihood of small vessel occlusion following intravenous injection [118, 119]. Second, exosomes are responsible for cell-to-cell transmission, and when brain injury occurs, endogenous NSCs release exosomes to convey the message of the need for rescue to other cells to achieve the repair and regeneration of the damaged brain. Third, with low immunogenicity, exosomes secreted by NSCs provide a cell-free therapy for neurological diseases without significant side effects [118]. Fourth, compared with the difficulty of stem cell culture and preservation, the risk of growth cannot be controlled after implantation, and due to the suspicion of tumour formation, stem cell exosomes can be stored in preparation and injected into the blood, where they are easily absorbed. In view of this, studying NSC exosomes provides insight into optimizing cell-based therapies and helps in understanding the exact mechanism of regeneration and repair based on exosome therapy after brain injury.

Exosomes secreted from NSCs have shown promise in the field of regenerative therapy, including the treatment of TBI. Despite the ever-increasing number and promising results of NSC-derived exosome studies in TBI therapy, translational research towards preclinical and clinical studies remains a long way off. There are still many barriers to overcome before they can be successfully implemented for TBI patients. As discussed in the review, these limitations include establishing a stable amplification system of NSC exosomes without heterologous components, the creation of uniform standards for purification and storage methods for the formation of NSC exosomes and, crucially, defining a method for single-exosome NSC analysis for a better understanding of the characteristics and mechanisms underlying exosome-based therapies.

Exosomes have obvious heterogeneity. One crucial reason is that their small size and limited carrying capacity result in the differential distribution of specific proteins. Another reason is that gene expression changes induced by environmental factors such as circadian rhythm, stimulation, stress, infection and cell cycle may drive the occurrence of exosome heterogeneity. Therefore, a suitable and effective method for the purification of NSC exosomes should be employed to meet the requirements of current good manufacturing practices without changing the biological activity of exosomes, which can effectively provide a homogeneous NSC exosome population containing similar RNA, proteins and lipids. Regarding exosome purification methods, although a variety of purification methods have been established, there are biological differences in the exosomes obtained by each method. Ensuring high yield and high quality of exosomes is the key to exosome research. At present, there is still a lack of safe, effective and high-yield methods for the production and purification of exosomes, which limits the clinical transformation of exosomes for disease treatment. For exosome preservation methods, exosomes are routinely resuspended in PBS or 10% DMSO and stored at − 80 °C, which does not ensure exosome activity. Therefore, the purification and preservation method standards of NSC exosomes should be thoroughly discussed to ensure the acquisition of high-yield and high-quality exosomes and to promote the clinical transformation of NSC exosomes for TBI treatment. Moreover, our understanding of the biological functional diversity of NSC exosomes is still limited, and the mechanism of interaction between NSC exosomes and recipient cells to regulate target cells cannot be drawn in depth. Consequently, the release, transport route and fate of NSC exosomes are tracked at the level of single vesicles combined with imaging methods, and the basic functions of exosomes are further elucidated for the theoretical foundation for future clinical transformation.

Despite the current challenges in the clinical translation of NSC exosomes, in addition to gene modification, NSC exosomes can also be combined with drug-carrying nanoparticles to realize the advantages of both and give NSC exosomes broader development prospects in the future.

## Conclusion

In summary, presently one of the most promising treatment approaches for brain injury is based on NSC exosome administration. Due to the beneficial effect on NSC exosomes after damage to nerves in the brain, it is necessary to explore the potential mechanisms in the regulation of neuroregeneration by NSC exosomes. The underlying mechanisms of exosomes derived from NSCs for brain injury are probably due to their neuroregeneration, anti-inflammatory, angiogenesis and anti-apoptosis properties (Fig. 3). Furthermore, considering that engineered exosomes of NSCs may affect brain function at different levels, the efficacy of TBI varies for different treatment routes (intravenous, intranasal or in situ). Therefore, tailoring NSC exosome therapy strategies according to the needs of TBI patients is crucial to enhance the effect of exosome-based cell-free therapy.

## Data Availability

Not applicable.

## Abbreviations

TBI: Traumatic brain injury
NSCs: Neural stem cells
SVZ: Subventracular zone
SGZ: Subgranular zone
BDNF: Brain-derived neurotrophic factors
GDNF: Glial cell line-derived neurotrophic factors
RNA: Ribonucleic acid
hNSCs: Human neural stem cells
ILVs: Intraluminal vesicles
MVBs: Multivesicular bodies
ESCRT: Endosomal sorting complex required for transport
Aβ: Amyloid β-protein
AD: Alzheimer’s disease
PD: Parkinson’s disease
MSCs: Mesenchymal stem cells
CAR: Coxsackie-adenovirus receptor
Ad5: Adenovirus type 5
Exo: Exosome
BB: Blood‒brain barrier
VEGF: Vascular endothelial growth factor
SCMECs: Spinal microvascular endothelial cells
Caspase: Cysteine-containing aspartate proteolytic enzyme
ROS: Reactive oxygen species
HUVECs: Human umbilical vein endothelial cells
mRNA: messenger RNA
miRNA: MicroRNA
LncRNA: Long noncoding RNA
Hes1: Hes family bHLH transcription factor 1
